# Haploinsufficiency of the *c-myc* transcriptional repressor *FIR*, as a dominant negative-alternative splicing model, promoted p53-dependent T-cell acute lymphoblastic leukemia progression by activating Notch1

**DOI:** 10.18632/oncotarget.3244

**Published:** 2014-12-31

**Authors:** Kazuyuki Matsushita, Kouichi Kitamura, Bahityar Rahmutulla, Nobuko Tanaka, Takayuki Ishige, Mamoru Satoh, Tyuji Hoshino, Satoru Miyagi, Takeshi Mori, Sakae Itoga, Hideaki Shimada, Takeshi Tomonaga, Minoru Kito, Yaeko Nakajima-Takagi, Shuji Kubo, Chiaki Nakaseko, Masahiko Hatano, Takashi Miki, Masafumi Matsuo, Masaki Fukuyo, Atsushi Kaneda, Atsushi Iwama, Fumio Nomura

**Affiliations:** ^1^ Department of Molecular Diagnosis, Graduate School of Medicine, Chiba University, Inohana, Chiba, Japan; ^2^ Division of Laboratory Medicine, Chiba University Hospital, Inohana, Chiba, Japan; ^3^ Department of Physical Chemistry, Graduate School of Pharmaceutical Sciences, Chiba University, Inohana, Chiba, Japan; ^4^ Department of Cellular and Molecular Medicine, Graduate School of Medicine, Chiba University, Inohana, Chuo-ku, Chiba, Japan; ^5^ Department of Pediatrics, Graduate School of Medicine, Kobe University, Kusunoki-cho, Kobe, Japan; ^6^ Department of Surgery, School of Medicine, Toho University, Omori-nishi, Ota-ku, Tokyo, Japan; ^7^ Laboratory of Proteome Research, National Institute of Biomedical Innovation, Saito-Asagi, Ibaraki, Osaka, Japan; ^8^ Oriental Yeast Co., Ltd. Azusawa, Itabashi-ku, Tokyo, Japan; ^9^ Department of Genetics, Hyogo College of Medicine, Mukogawa-cho, Nishinomiya, Hyogo Prefecture, Japan; ^10^ Department of Haematology, Chiba University Hospital, Inohana, Chiba, Japan; ^11^ Department of Biomedical Science, Graduate School of Medicine, Chiba University, Inohana, Chiba, Japan; ^12^ Department of Medical Physiology, Graduate School of Medicine, Chiba University, Inohana, Chiba, Japan; ^13^ Department of Medical Rehabilitation, Faculty of Rehabilitation, Kobegakuin University, Arise, Ikawadani, Nishi, Kobe, Japan; ^14^ Department of Molecular Oncology, Graduate School of Medicine, Chiba University, Inohana, Chiba, Japan

**Keywords:** FBP interacting repressor (FIR), splicing variant, haplo-insufficiency, leukemia, P53, T-ALL

## Abstract

FUSE-binding protein (FBP)-interacting repressor (FIR) is a *c-myc* transcriptional suppressor. A splice variant of FIR that lacks exon 2 in the transcriptional repressor domain (FIRΔexon2) upregulates *c-myc* transcription by inactivating wild-type FIR. The ratio of *FIRΔexon*2/*FIR* mRNA was increased in human colorectal cancer and hepatocellular carcinoma tissues. Because FIRΔexon2 is considered to be a dominant negative regulator of FIR, FIR heterozygous knockout (*FIR*^+/−^) C57BL6 mice were generated. FIR complete knockout (*FIR*^−/−^) was embryonic lethal before E9.5; therefore, it is essential for embryogenesis. This strongly suggests that insufficiency of FIR is crucial for carcinogenesis. *FIR*^+/−^ mice exhibited prominent *c-myc* mRNA upregulation, particularly in the peripheral blood (PB), without any significant pathogenic phenotype. Furthermore, elevated *FIRΔexon2*/*FIR* mRNA expression was detected in human leukemia samples and cell lines. Because the single knockout of *TP53* generates thymic lymphoma, *FIR*^+/−^*TP53*^−/−^ generated T-cell type acute lymphocytic/lymphoblastic leukemia (T-ALL) with increased organ or bone marrow invasion with poor prognosis. RNA-sequencing analysis of sorted thymic lymphoma cells revealed that the Notch signaling pathway was activated significantly in *FIR*^+/−^*TP53*^−/−^ compared with that in *FIR*^+/+^*TP53*^−/−^ mice. *Notch1* mRNA expression in sorted thymic lymphoma cells was confirmed using qRT-PCR. In addition, flow cytometry revealed that *c-myc* mRNA was negatively correlated with *FIR* but positively correlated with *Notch1* in sorted T-ALL/thymic lymphoma cells. Moreover, the knockdown of *TP53* or *c-myc* using siRNA decreased Notch1 expression in cancer cells. In addition, an adenovirus vector encoding FIRΔexon2 cDNA increased bleomycin-induced DNA damage. Taken together, these data suggest that the altered expression of *FIRΔexon2* increased Notch1 at least partially by activating c-Myc via a TP53-independent pathway. In conclusion, the alternative splicing of FIR, which generates FIRΔexon2, may contribute to both colorectal carcinogenesis and leukemogenesis.

## INTRODUCTION

DNA damage affects carcinogenesis, transcription, alternative splicing, and cell cycle control; however, the precise mechanism behind these affects remains largely unexplored. FUSE-binding protein (FBP) is a transcription factor that stimulates *c-myc* expression [1-3]. FBP-interacting repressor (FIR) is a *c-myc* transcriptional repressor that functions by suppressing the TFIIH/P89/XPB helicase (P89) [4-7]; hence, enhanced FIR showed antitumor effect in mouse xenografted model by suppressing *c-myc* [8-10]. Markedly, a splice variant of FIR that lacks exon 2 in the transcriptional repression domain (FIRΔexon2) elevates c-Myc protein expression *in vitro* [11]. FIRΔexon2 mRNA is frequently upregulated in human colorectal cancers [12] as well as hepatocellular carcinoma [13], where it stimulates tumor growth by preventing FIR from suppressing *c-myc* [13]. FIRΔexon2 functions as a dominant negative regulator of FIR; therefore it reduces FIR function. Recent studies suggested that DNA damage induces alternative splicing of several genes including *FIR* [14,15]. Specifically, FIR/FIRΔexon2 monitors the DNA damage response by potentially interacting with DNA-PKcs or Ku-86 [14]. Therefore, DNA damage may induce persistent *c-myc* upregulation via FIRΔexon2 in cancer cells, whereas it induces TP53 in normal cells

FIR is a splice variant of PUF60, reported as a splicing factor that lacks the exon 5 consists of 17 amino acids [16]. SAP155, a subunit of the SF3b spliceosome complex, interacts directly with PUF60 *in vitro* [17] and could be co-immunoprecipitated with FIR (or FIRΔexon2)-FLAG beads *in vivo* [18]. Furthermore, SAP155 is required for proper FIR pre-mRNA splicing; therefore, SAP155-FIR complex formation inhibits the well-established functions of both SAP155 and FIR, disturbing splicing and the transcriptional suppression of *c-myc* [18,19]. Accordingly, the FIR/FIRΔexon2/SAP155 interaction, which affects *FIR* and *p21Kip1* splicing, links the DNA damage response to *c-myc* regulation [19]. In fact SAP155 mutations, which potentially affect FIR/FIRΔexon2/SAP155 formation, were reported not only in myeloid lineage tumors but also lymphoid lineage tumors [20-23]. Consequently, an aberrant FIR/FIRΔexon2/SAP155 interaction is responsible for cancer development and differentiation and is a potent target for cancer screening and treatment [13, 19].

The upregulation of c-Myc and Notch1 with TP53 loss-of-function is critical for T-ALL pathogenesis [24]. This mechanism involves the loss of F-box WD repeat-containing protein 7 (FBW7/FBWX7), which was reported to induce sustained c-Myc and Notch1 expression via a post-transcriptional mechanism, resulting in TP53-deficient T-ALL [25, 26]. FBW7 is required for the polyubiquitination-mediated proteasomal degradation of c-Myc. Accordingly, FBW7 modulates leukemia-initiating cell (LIC) activity by regulating c-Myc stability [25], and thereby plays a role in the pathogenesis [26]. However, the mechanism of c-Myc upregulation in T-ALL in the absence of *TP53*, *FBW7*, or *Notch1* mutations is unclear. In this study, the significance of disturbed *FIR* expression was examined by generating *FIR*^+/−^ mice to assess the dominant negative effect of FIRΔexon2. This study indicated that the alternative splicing of FIR links the DNA damage response to *c-myc* regulation and revealed how the alteration of FIR affects c-Myc, Notch1, or TP53 during the pathogenesis of T-ALL in a *FIR*^+/−^*TP53*^−/−^ mouse model.

## RESULTS

### *FIR*^−/−^ mice were embryonic lethal at E9.5 or earlier

**Figure 1:**
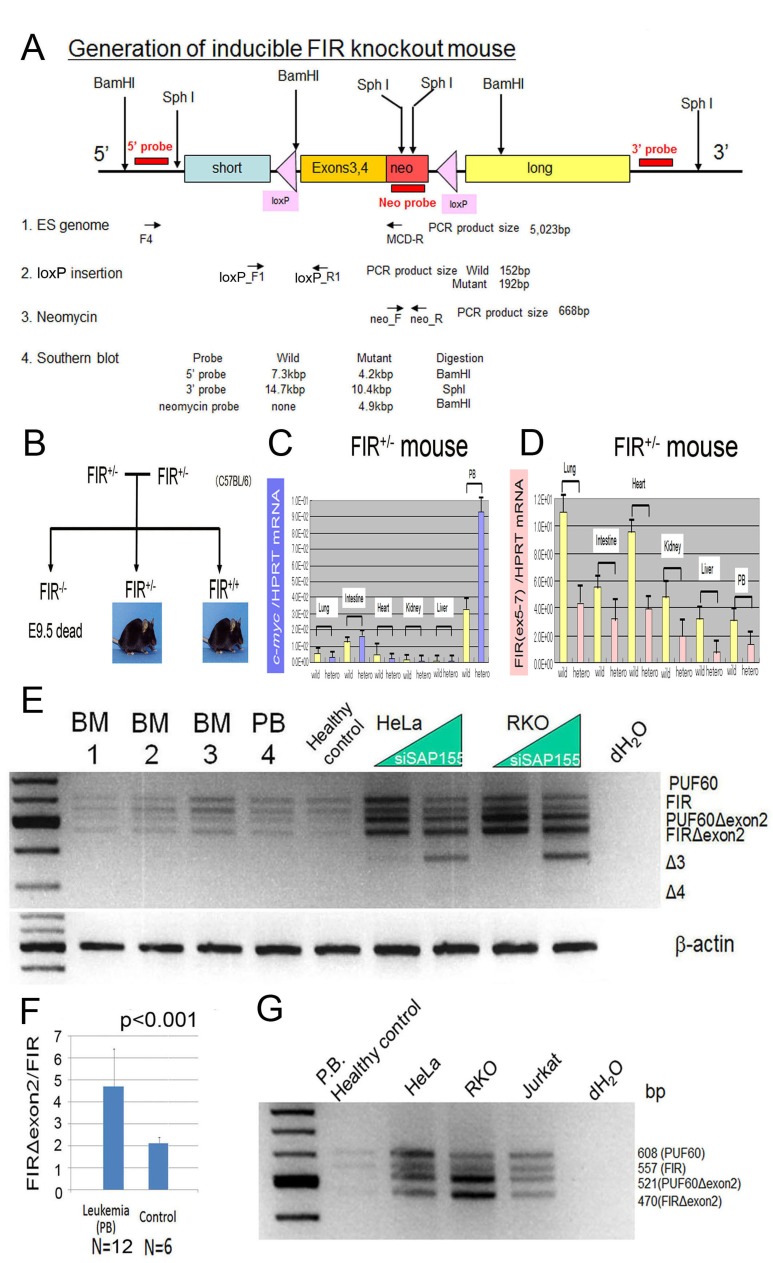
c-Myc mRNA was activated in the peripheral blood cells of inducible FIR heterozygous knockout mouse and FIR/IRΔexon2 mRNA expression in human clinical leukemia/malignant lymphoma samples (A) Genetic construction for inducible *FIR*^+/−^ mouse. Primers locations for detecting ES genome, LoxP insertion, neomycin cassette and probes for Southern blot analysis were indicated. Expected DNA sizes for Southern blot analysis are also shown. (B) Family tree to obtain FIR homo knockout mouse *FIR*^−/−^, by cross-fertilization between FIR hetero knockout mouse: *FIR*^+/−^. *FIR*^−/−^ mouse was revealed to be embryonic lethal at least before E9.5 by cross-fertilized between FIR hetero knockout mouse. The relative expression of *c-myc* and FIR mRNAs from lung, intestinal mucosae, heart muscle, kidneys, livers and peripheral blood (PB) were examined. (C) *c-myc* mRNA of PB of FIR hetero knockout mice was three-times higher than those of wild mouse. (D) FIR mRNA expression level of FIR hetero knockout mice was exactly half of those of wild mouse. (E) qRT-PCR of PUF60, FIR PUF60Δexon2, FIRΔexon2 mRNA were indicated by RT-PCR. Samples: leukemia cells from peripheral blood (PB) and bone marrow (BM) of adult patients (listed in table 2). (F) The ratio of FIR/FIRΔexon2 mRNA level of leukemia cells was significantly higher than those of non-leukemia or control samples (Student's t-test). (G) mRNA extracted from HeLa (human cervical squamous carcinoma cells), RKO (human colon adenocarcinoma cells), and Jurkat (human immortalized T lymphocyte) cells was examined for their FIR splicing variants expression.

The design of the FIR-targeting vector (Figure 1A), as well as the wild-type, targeted, and deleted *FIR* alleles used to prepare the *FIR*^+/−^ mice are shown in Figure S1. The *FIR* homozygous knockout mouse *FIR*^−/−^ *was* prepared by the cross-fertilization of *FIR*^+/−^ mice (Figure 1B). A total of 86 mice were analyzed after the genetically confirmed mating of *FIR*^+/−^ mice. *FIR*^+/−^ and *FIR*^+/+^ were recovered at close to the expected Mendelian ratio of 48:26 (~2:1; Table 1). There were 12 dead embryos (hypothetically *FIR*^−/−^): six between E13.5 and E14.5 and six on E9.5 (Table 1). No live *FIR*^−/−^ mice were observed at birth, E13.5–E14.5, or E9.5 (Figure 1B, Table 1). *FIR*^−/−^ mice exhibited early developmental defects and die by E4.5 or earlier (Dr. David Levens, NCI, USA). *FIR* total knockout, *FIR*^−/−^*,* mouse is embryonic lethal before E9.5, suggesting that FIR is essential for embryogenesis. Proteins expressed during embryogenesis disappear during development but are re-expressed in cancers [27, 28], suggesting that FIR is crucial for carcinogenesis as well.

**Table 1:** The number of FIR hetero and homo knockout mice during the time of observation

	*FIR*^+/+^	*FIR*^+/−^	*FIR*^−/−^	supposed *FIR*^−/−^	
Time at obervation	Wild mice	FIR hetero knockout mice	FIR homo knockout mice	Dead embryo	Total
At birth	12	17	0	0	29
E13.5 to E14.5	9	20	0	6	35
E9.5	5	11	0	6	22
Total	26	48	0	12	86

### *FIR*^+/−^ mice exhibited increased *c-myc* mRNA expression but had no significant deleterious phenotype

The relative expression of *c-myc* (Figure 1C and *FIR* (Figure 1D) mRNA in the lungs, intestines, heart, kidney, liver, and peripheral blood (PB) of *FIR*^+/−^ mouse was approximately half of that detected in wild-type mice. However, *FIR*^+/−^ mouse had no apparent pathogenic phenotype. Recently, five individuals were reported with *de novo* interstitial 8q24.3 deletions ranging from 65 kb to 1 Mb on the chromosome that includes FIR (PUF60). These deletions had a clinical phenotype that was associated with multiple systemic phenotypes but no hematological malignancy or lymphoma [29, 30]. This suggests that the haploinsufficiency of *PUF60* (*FIR*) with *c-myc* mRNA elevation alone is not sufficient to drive the pathogenesis of T-ALL.

### FIR is alternatively spliced in human leukemia

To explore how *c-myc* is activated in T-ALL/lymphoma, the alternative splicing of FIR, the ratio of FIR/FIRΔexon2, and *c-myc* mRNA expression were examined in human leukemia samples (Figure 1E, Table S1). qRT-PCR for the cDNA of full-length FIR variants was performed in bone marrow or peripheral blood samples using primers to amplify the amino terminal region. At least four variants (FIR, PUF60, FIRΔexon2, and PUF60Δexon2) were expected from the alternative use of the two potential exons [12]. The ratio of *FIR*/*FIRΔexon2* mRNA (Figure 1F) was significantly higher in leukemia cells compared with that in non-leukemia or control samples from adults (Figures 1G) and children (data not shown). This suggests that the alternative splicing of *FIR* and the ratio of *FIR*/*FIRΔexon2* may contribute to *c-myc* upregulation in *T-ALL*. Notably, c-Myc upregulation alone by FIR haploinsufficiency did not generate leukemia. Thus, *FIR* splicing variants rather than *FIR* haploinsufficiency significantly contributes toward promoting the progression of T-ALL/lymphoma via a c-Myc-independent pathway.

### *FIR*^+/−^*TP53*^−/−^ promotes the bone marrow invasion of T-cell malignant lymphoma

**Figure 2:**
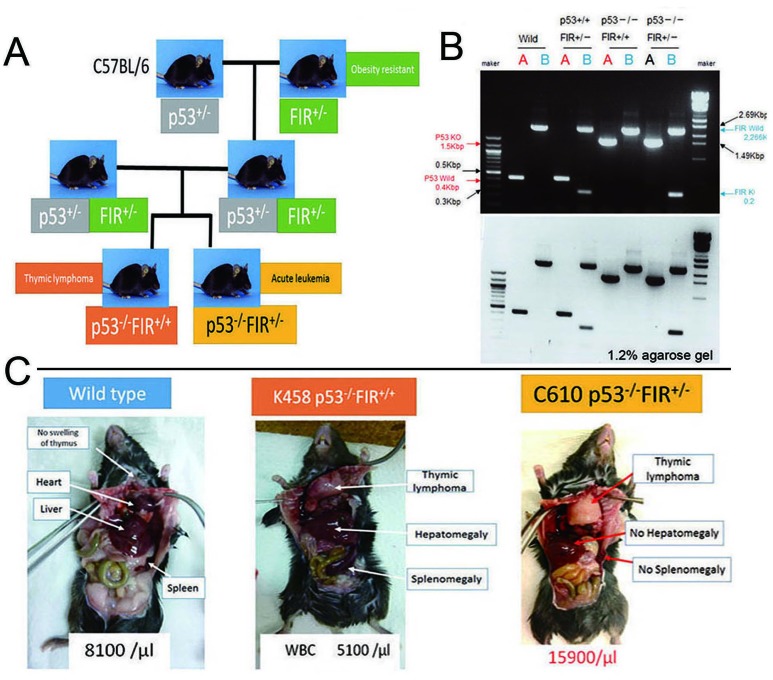
Preparation of *FIR*^+/−^*P53*^−/−^ and *FIR*^+/+^*P53*^−/−^ mouse (A) *FIR*^+/−^ and *P53*^−/−^ were obtained by cross-fertilization between *FIR*^+/−^*P53*^−/−^ and *FIR*^+/+^*P53*^−/−^ mice. *FIR*^+/−^*P53*^+/−^ double compound hetero knockout mouse was prepared and mated each other, or female *P53*^+/−^ was mated with male *FIR*^+/−^*P53*^+/−^ mouse to obtain *FIR*^+/−^*P53*^−/−^ because *FIR*^+/−^*P53*^+/−^ showed low fertility. (B) Genotyping of *FIR*^+/+^*P53*^−/−^, *FIR*^+/−^*P53*^+/+^ and *FIR*^+/−^*P53*^−/−^ and wild mice were confirmed by PCR. (C) Thymic lymphoma was observed in *FIR*^+/+^*p53*^−/−^ mice.

**Figure 3:**
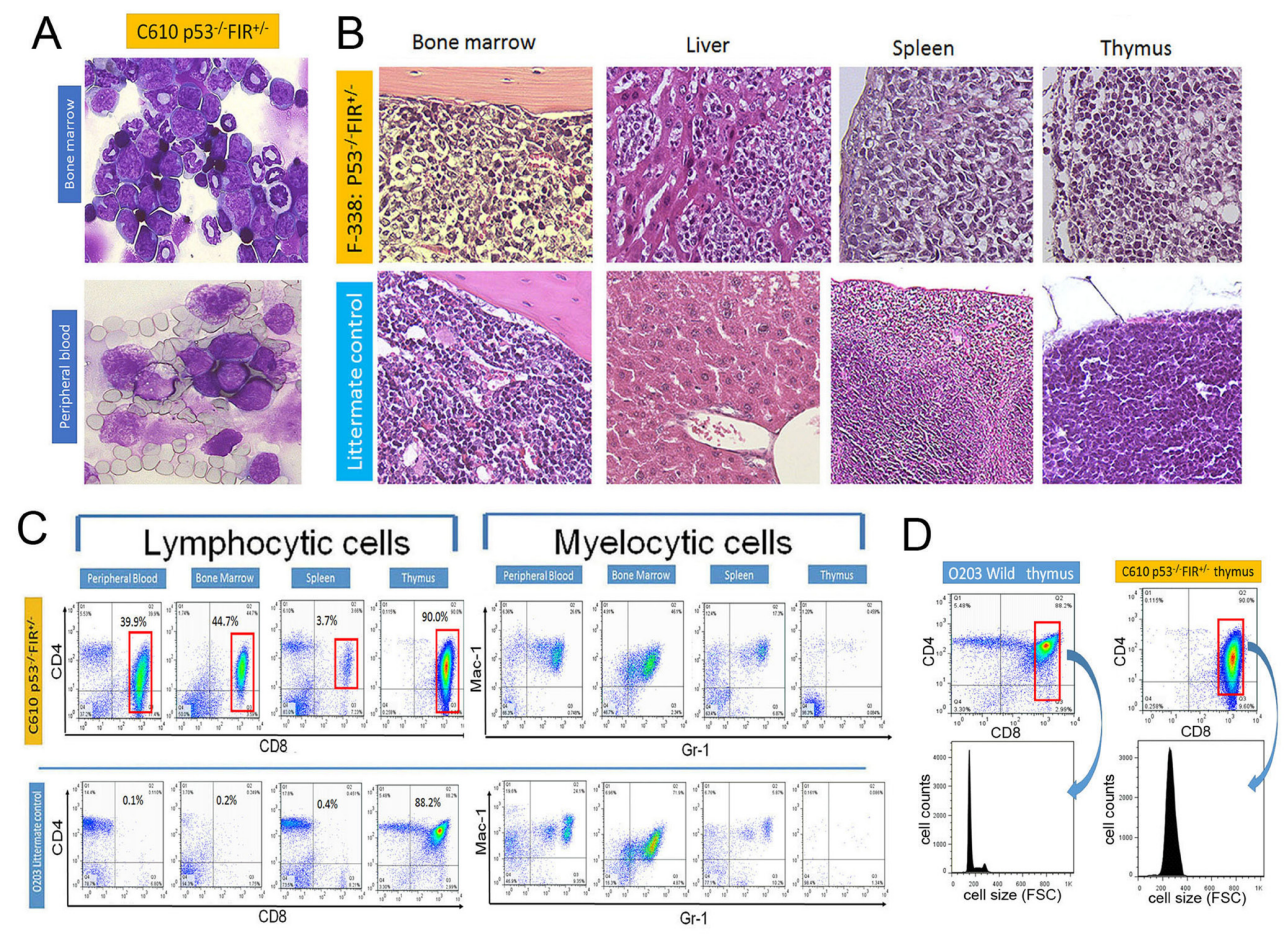
Histologic features and flow cytometry analysis of *FIR*^+/−^*P53*^−/−^ (A) Atypical cells were indicated by Giemsa stain in bone marrow and peripheral blood of *FIR*^+/−^*P53*^−/−^ mouse (C610). (B) Histologic features of bone marrow, liver, spleen and thymus in *FIR*^+/−^*P53*^−/−^ (F338) and wild mouse *by* Hematoxylin-Eosin *stain*. (C) Flow cytometry analysis of lymphocytic cells with CD4 and CD8 as indicated markers. Mac1 and Gr1 were used for myelocytic markers. Flow cytometry analysis revealed that lymphocytic atypical cells (left) were CD4^low+^CD8^+^ phenotype (gated area) but no significant findings in myeloid cells (right) in *FIR*^+/−^*P53*^−/−^ mouse (C610), and diagnosed as T-cell type acute lymphocytic/lymphoblastic leukemia (T-ALL)/lymphoma. (D) Cell size of gated area was measured by flow cytometry analysis (FSC: Forward Scatter).

Because the *FIR*^+/−^ mice suggested significant *c-myc* upregulation in the PB without a significant pathogenic phenotype, we generated *FIR*^+/−^*TP53*^+/−^ double compound heterozygous knockout mice and cross-fertilized or mated female *TP53*^+/−^ with male *FIR*^+/−^*TP53*^+/−^ mice because of the low fertility of *FIR*^+/−^*TP53*^+/−^ mice (Figure 2A). The genotypes of the *FIR*^+/−^*TP53*^−/−^ and *FIR*^+/+^*TP53*^−/−^ mice were confirmed by PCR analysis of genomic DNA (Figure 2B). *FIR*^+/−^*TP53*^−/−^ and *FIR*^+/+^*TP53*^−/−^ mice were sacrificed when a loss of 10%–15% bodyweight during growth or a systemic disorder such as dyspnea with a loss of movement was observed. A demonstrative macroscopic view of the *organs of FIR*^+/−^*TP53*^−/−^*, FIR*^+/+^*TP53*^−/−^, and wild-type mice are shown in Figure 2C. Atypical cells in *FIR*^+/−^*TP53*^−/−^ mice that were observed in the PB, bone marrow (BM), liver, spleen, and thymus are shown in Figures 3A and B. Next, flow cytometry analysis of the PB, spleen, thymus, and BM of thymic lymphoma of *FIR*^+/+^*TP53*^−/−^ mice was performed (Figure 3C). These analyses revealed that both *FIR*^+/+^*TP53*^−/−^ *and FIR*^+/−^*TP53*^−/−^ mice exhibited T-ALL/T-cell-type thymic lymphoma. Therefore, the single knockout of *TP53*^−/−^ was sufficient to cause T-ALL/T-cell-type thymic lymphoma. Flow cytometry also revealed that the size of the thymic lymphoma cells was apparently larger in *FIR*^+/−^*TP53*^−/−^ *mice* compared with that in control mice (Figure 3D, gated area).

### Haploinsufficiency of *FIR* developed rapid T-ALL progression with bone marrow invasion

**Figure 4:**
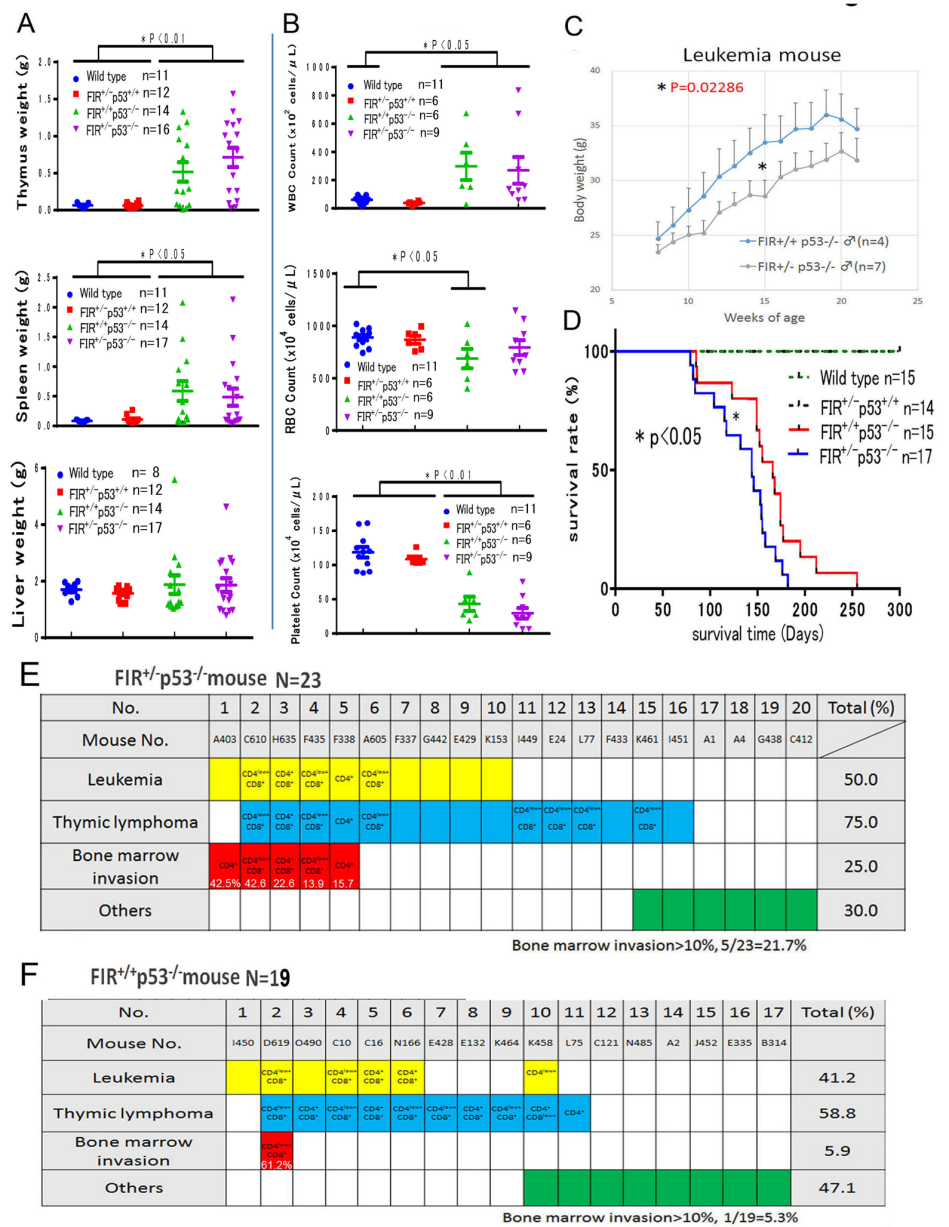
Summary of organs' weight and blood tests, body weight and overall survival of curves of *FIR*^−/−^*P53*^+/+^, *FIR*^+/−^*P53*^+/+^, *FIR*^+/+^*P53*^−/−^, and *FIR*^+/−^*P53*^−/−^ mice (A) The weight of thymus of *FIR*^+/−^*P53*^−/−^ *and FIR*^+/+^*P53*^−/−^ was significantly heavier than that of wild or *FIR*^+/−^*P53*^+/+^ mouse (P<0.05). The weight of spleen of *FIR*^+/+^*P53*^−/− -^were significantly heavier than that of wild or *FIR*^+/−^*P53*^+/+^ mouse (P<0.05). The weight of liver of wild mouse, *FIR*^+/−^*P53*^+/+^, *FIR*^+/+^*P53*^−/−^, and *FIR*^+/−^*P53*^−/−^ was no significant difference. (B) WBC count of *FIR*^+/−^*P53*^−/−^ *and FIR*^+/+^*P53*^−/−^ was significantly increased than that of wild mouse. RBC count of *FIR*^+/+^*P53*^−/−^ was significantly less than that of wild mouse (P<0.05). Platelet count of *FIR*^+/−^*P53*^−/−^ *and FIR*^+/+^*P53*^−/−^ was significantly less than that of wild or *FIR*^+/−^*P53*^+/+^ mouse (P<0.05). (C) *Body weight of FIR*^+/−^P53^−/−^ mice was significantly lighter than that of *FIR*^+/+^*P53*^−/−^. Statistical signifincace was calculated by Student's t-test. (D) The overall survival curves of four genetically different group: wild, *FIR*^+/−^*p53*^+/+^, *FIR*^+/−^*p53*^−/−^, and *FIR*^+/+^*p53*^−/−^ mice. *FIR*^+/−^*p53*^+/+^ and *FIR*^+/+^*p53*^+/+^ were survived 100% up to 25 weeks after birth without obvious tumor formation, body weight loss or other physical disabilities. On the contrary, the overall survival curves (Kaplan-Meier method) of *FIR*^+/−^*p53*^−/−^ and *FIR*^+/+^*p53*^−/−^ mice were declined around 70 days after birth. Overall survival curves of four genetically different group: wild, *FIR*^+/−^*p53*^+/+^, *FIR*^+/−^*p53*^−/−^, and *FIR*^+/+^*p53*^−/−^ mice were compared by log-rank test. (E) T-ALL with more than 10 % bone marrow infiltration of blast cells in *FIR*^+/−^*P53*^−/−^ *mice was* 5 out of 23 (21.7%) including three pre-analytical alive mouse *in FIR*^+/+^*P53*^−/−^ *(*1 out of 19=5.3%) including two pre-analytical alive mouse. (F) In *FIR*^+/−^*P53*^−/−^ *mice (N=23),* T-ALL*: 10 (50.0%), thymic lymphoma: 15 (75%), bone marrow invasion: 5 (25.0%). Whereas in FIR*^+/+^*P53*^−/−^ mice (N=17) T-ALL: 7 (41.2%), thymic lymphoma: 10 (58.8%), bone marrow invasion: 1 (5.9%). Blank in colored column indicated undetermined or not tested for cell surface marker.

The weight of the thymus was significantly heavier in *FIR*^+/−^*TP53*^−/−^ *and FIR*^+/+^*TP53*^−/−^ mice compared with that in wild-type or *FIR*^+/^^−^*TP53*^+/+^ mice. In addition, the weight of the spleen was significantly heavier in *FIR*^+/+^*TP53*^−/−^ mice than that in wild-type or *FIR*^+/−^*TP53*^+/+^ mice. The WBC count was increased significantly in *FIR*^+/−^*TP53*^−/−^ *and FIR*^+/+^*TP53*^−/−^ mice compared with that in wild-type. Conversely, the RBC count was significantly lower in *FIR*^+/+^*TP53*^−/−^ compared with that in wild-type mice. The platelet count was also significantly lower in *FIR*^+/−^*TP53*^−/−^ *and FIR*^+/+^*TP53*^−/−^ mice compared with that in wild-type or *FIR*^+/−^*TP53*^+/+^ mice (Figures 4A, B). *FIR*^+/−^*TP53*^−/−^ mice with T-ALL/lymphoma exhibited a significantly lower bodyweight than did *FIR*^+/+^ (Figure 4C). One-hundred percent of wild-type and *FIR*^+/−^*TP53*^−/−^ mice survived during the study period, and the overall survival rate (*Kaplan–Meier)* of *FIR*^+/+^*TP53*^−/−^ mice was better than that of *FIR*^+/−^*TP53*^−/−^ (Figure 4D). There were no significant differences in the nose-to-anus length and bodyweight of *FIR*^+/−^ compared with that of wild-type mice (*FIR*^+/+^) (Figure S2). These results suggest that *FIR* haploinsufficiency promoted the progression of T-ALL/lymphoma, reduced bodyweight, and was associated with a poorer prognosis. The incidence of T-ALL with > 10% bone marrow infiltration of blast cells was higher in *FIR*^+/−^*TP53*^−/−^ mice (5 of 23; 21.7%, including three live mice before analysis) compared with that in *FIR*^+/+^*TP53*^−/−^ (1 of 19; 5.3%, including two live mice before analysis) (Figures 4E, F). These results demonstrated that high levels of c-Myc promoted an increased occurrence of T-ALL and bone marrow infiltration in this mouse model. Of the *FIR*^+/−^*TP53*^−/−^ mice (N = 20), 10 had T-ALL (50.0%), 15 had thymic lymphoma (75%), and five experienced bone marrow invasion (25.0%). In contrast, of the *17 FIR*^+/+^*P53*^−/−^ mice*, seven had* T-ALL (41.2%), 10 had thymic lymphoma (58.8%), and one exhibited bone marrow invasion (5.9%) (Figure 4F).

### Comparison of RNA-sequencing analysis of sorted thymic lymphoma cells from *FIR*^+/−^*TP53*^−/−^ or *FIR*^+/+^*TP53*^−/−^ mice

**Figure 5:**
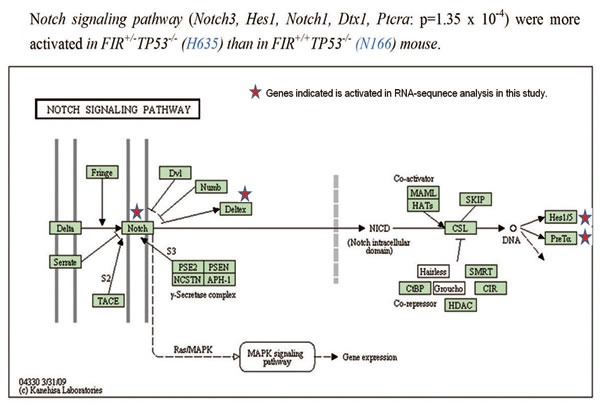
Signaling pathway activated in sorted thymic lymphoma cells in *FIR*^+/−^*TP53*^−/−^ mice (A) KEGG pathway analysis showed that Notch Signaling pathway was more activated in *FIR*^+/−^*TP53*^−/−^ mice compared with *FIR*^+/−^*TP53*^−/−^ and *FIR*^+/+^*TP53*^−/−^ mice, with the *Notch3*, *Hes1*, *Notch1*, *Dtx1* and *Ptcra* genes upregulated (P=1.4×10^−4^). Other activated pathways were also shown in Figure S6.

RNA-sequencing was used to compare the gene expression profiles of sorted thymic lymphoma cells from FIR^+/−^*TP53*^−/−^ and *FIR*^+/+^*TP53*^−/−^ mice. The top 100 activated genes were analyzed (Figure S3A), and data revealed that the Notch signaling pathway was activated more in CD4^+^CD8^+^ *t*hymic lymphoma cells from *FIR*^+/−^*TP53*^−/−^ mice compared with those from *FIR*^+/+^*TP53*^−/−^mice(Table S3A–C). RNA-sequencing analysis of sorted *CD4*^+^*CD8*^+^ thymic lymphoma cells revealed that the Notch signaling (Figure 5) and tight junction pathways (Figure S4A) were activated more significantly in *FIR*^+/−^*TP53*^−/−^ (H635) than *FIR*^+/+^*TP53*^−/−^ (N166) mice. In contrast, analysis of *CD4*^low+^*CD8*^+^ thymic lymphoma cells demonstrated that the focal adhesion pathway (Figure S4B*)* was activated more significantly in *FIR*^+/−^*TP53*^−/−^ (A605) than in *FIR*^+/+^*TP53*^−/−^ (D619) *mice.* The upregulation of *c-myc* and *notch1* mRNA was confirmed by qRT-PCR (Table S4, Figure S3C–E). *Notch1* mRNA was more activated in both CD4^+^CD8^+^ and *CD4*^low+^*CD8*^+^ thymic lymphoma cells from *FIR*^+/−^*TP53*^−/−^ *mice* compared with those from *FIR*^+/+^*TP53*^−/−^ *mice* (Figure S3C, D). In contrast, *c-myc* mRNA was activated in whole peripheral blood cells in two *FIR*^+/−^*TP53*^+/+^ *mice* examined in this study (Figure S3E).

### c-Myc and FIR proteins were negatively correlated and *c-myc* and *notch1* mRNAs were positively correlated in sorted thymic lymphoma/T-ALL cells

**Figure 6:**
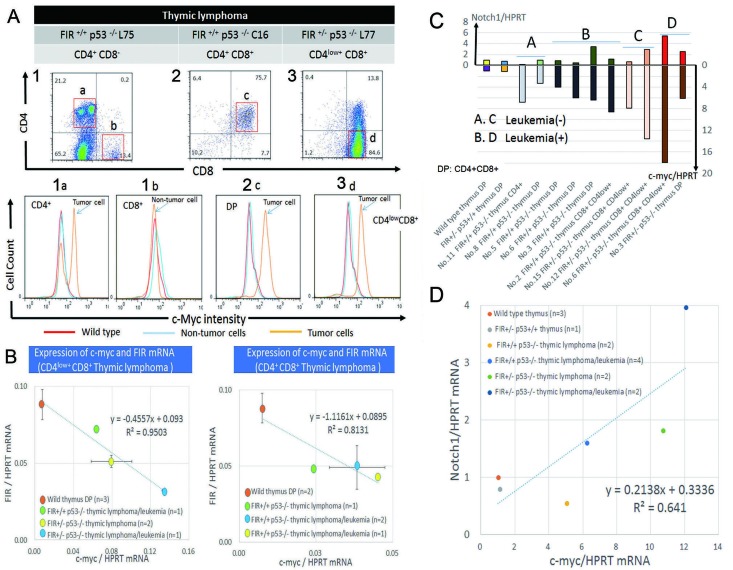
c-Myc protein is enhanced and showed inverse correlation with FIR in thymic lymphoma cells and promotes bone marrow invasion (A) Flow cytometry of thymic lymphoma cells in *FIR*^+/+^*P53*^−/−^ or *FIR*^+/−^*P53*^−/−^ mice are indicated. CD4^+^ cells of *FIR*^+/+^*P53*^−/−^ (upper column 1) has two populations indicated c-Myc-high (1a) and c-Myc–low intensity (1b). Notably, c-Myc-high (1a) population, presumably lymphoma cells, has c-Myc-low peak, indicating non-tumor cells cluster. CD4^+^CD8^+^ cells (2c) also indicated c-Myc-high intensity population. CD4^low+^CD8^+^ (3d) showed c-Myc-high intensity population indicated by FACS (bottom). Red line: Thymus cells of wild type *FIR*^+/+^*P53*^+/+^ mouse. Thin blue line: non-tumor cells of *FIR*^+/+^*P53*^−/−^ or *FIR*^+/−^*P53*^−/−^ mice. Orange line: Tumor cells of *FIR*^+/+^*P53*^−/−^ or *FIR*^+/−^*P53*^−/−^ mice. (B) Inverse correlation with significance between *c-myc* and FIR mRNA expression in CD4^low+^CD8^+^ or CD4^+^CD8^+^thymus lymphocytes obtained from *FIR*^+/+^*P53*^−/−^ or *FIR*^+/−^*P53*^−/−^ mice. Relative *c-myc* (or FIR)/HPRT mRNA expression of CD4^low+^CD8^+^ thymic lymphoma/leukemia cells of *FIR*^+/+^*p53*^−/−^ (light green) was 8.6 (0.81), thymic lymphoma cells of *FIR*^+/−^*p53*^−/−^ (yellow) was 10.7 (0.58), and thymic lymphoma/leukemia cells of *FIR*^+/−^*p53*^−/−^ (blue) was 18.0 (0.37) times as compare to thymic cells of wild mouse (dark orange), respectively (right). The relationship between *c-myc*/HPRT mRNA (x-axis) and FIR/HPRT mRNA (y-axis) was y=-0.4557x+0.093 (R^2^=0.9503). Relative *c-myc* (or FIR)/HPRT mRNA expression of CD4^+^CD8^+^ thymic lymphoma cells of *FIR*^+/+^*p53*^−/−^ (light green) was 3.3 (0.56), thymic lymphoma/leukemia cells of *FIR*^+/+^*p53*^−/−^ (yellow) was 6.1 (0.48), and thymic lymphoma/leukemia cells of FIR^+/+^p53^−/−^ (blue) was 5.2 (0.56) times as compare to thymic cells of wild mouse (dark orange), respectively. The relationship between *c-myc*/HPRT mRNA (x-axis) and FIR/HPRT mRNA (y-axis) was y=-1.1161x+0.086 (R^2^=0.8131). (C) (D) c-myc mRNA and Notch1 mRNA expression was positively correlated each other in sorted thymic lymphoma cells extracted from mice of different genetic backgrounds.

Because FIR is believed to be a *c-myc* transcriptional repressor, c-Myc expression was examined in atypical or lymphoma cells from *FIR*^+/−^*P53*^−/−^ mice. As expected, c-Myc expression was higher in atypical/lymphoma cells compared with that in non-atypical cells or normal lymphocytes (Figure 6A). Furthermore, there was a significant negative correlation between *c-myc* and *FIR* mRNA expression in CD4^+^CD8^+^ and CD4^low+^CD8^+^ thymic lymphoma cells (Figure 6B. These results strongly suggest that FIR suppresses *c-myc* expression both *in vitro* and *in vivo*. Notably, atypical/lymphoma cells from *FIR*^+/+^*TP53*^−/−^ mice also expressed high levels of c-Myc. These results also suggest that activated *c-myc* mRNA, which does not directly reflect an increase in c-Myc protein, is inadequate for the pathogenesis of T-ALL. Therefore, the pathogenesis of T-ALL/lymphoma observed in *FIR*^+/+^*P53*^−/−^ mice was at least partly *Notch1* upregulation because *c-myc* and *Notch1* mRNAs were positively correlated (Figures 6C, D). Together, the current FIR haploinsufficiency mouse model revealed that T-ALL/lymphoma was generated via a p53-dependent pathway, but that its progression was potentially due to a c-Myc-independent mechanism because the *TP53* single knockout alone exhibited sustained c-Myc expression.

### Knocking down *TP53* and *c-myc* using siRNA suppressed Notch1 expression

**Figure 7:**
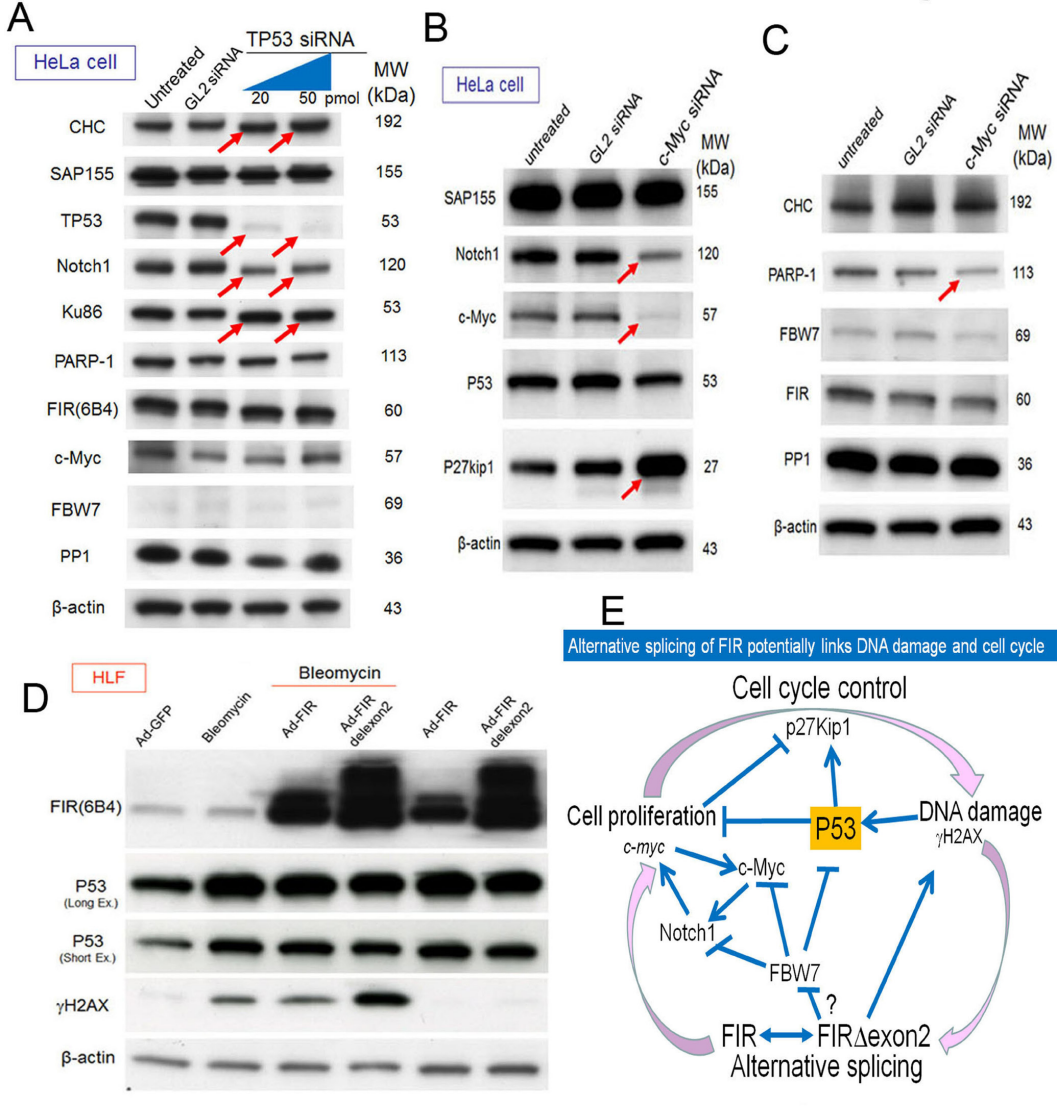
Alternative splicing of FIR connects DNA damage response, *c-myc* activation and cell cycle control (A) 20 and 50pmol of TP53 siRNA were transfected into HeLa cells. GL2 siRNA was transfected as the negative control. After 72h of incubation, whole-cell extracts were analyzed by western blotting for relevant protein expressions. (B, C) 20pmol c-Myc siRNA was transfected into HeLa cells. GL2 siRNA is for negative control. After 48h of incubation, whole-cell extracts were analyzed by western blotting for relevant protein expressions. (D) 3.76 × 10^8^ VP/ml (10 MOI) of Ad-FIR or Ad-FIRΔexon2 adenovirus vectors and DNA damaging agent bleomycin (30 μg/ml) were either co-treated or single treated into HLF cell. 3.76 × 10^8^ VP/ml (10 MOI) of GFP adenovirus (Ad-GFP) was treated as negative control. After 48h of incubation, whole-cell extracts were analyzed by western blotting. Severity of the DNA damage caused by bleomycin treatment is indicated by γH2AX expression. (E) Schematic view of haploinsufficiency, as a dominant negative-alternative splicing model of FIR in T-ALL pathogenesis. DNA damage affects alternative splicing of FIR that contributes to *c-myc* transcriptional activations. Activated c-Myc accelerates cell cycle by suppressing P27Kip1 and in turn accumulates DNA damage. The altered expression of FIRΔexon2 increased Notch1 at least partially by activating c-Myc via a TP53-independent pathway.

Bleomycin (BLM)-induced DNA damage induces FIR splicing [14]. The alternative splicing of *FIR* contributes to the transcriptional regulation of *c-myc*, which is critical for cell cycle control. Because Notch1 expression is pivotal for the pathogenesis of T-ALL, *TP53* or *c-myc* were knocked down using siRNA whereas BLM was treated as DNA damaging agent to examine the relationship among DNA damage, the alternative splicing of *FIR*, and cell cycle control. Knocking down both *TP53* and *c-myc* using siRNA significantly suppressed Notch1 expression without disturbing FIR expression (Figures 7A-C, arrows). TP53 expression was not affected significantly by the enforced expression by FIR//FIRΔexon2 using an adenovirus vector (Figure 7D). DNA damage affects the alternative splicing of FIR, which contributes to the transcriptional activation of *c-myc* via a dominant negative effect on endogenous FIR [14]. Activated c-Myc accelerates the cell cycle by suppressing p27Kip1 expression, which leads to the accumulation of DNA damage [14, 15]. These results suggest that disturbed *FIR* expression or the altered splicing of *FIR* may contribute to the pathogenesis of T-ALL via upregulating c-Myc-Notch1 axis independent on TP53 (Figure 7E). Therefore, *FIR* splicing is a novel mechanism that links DNA damage to *c-myc* regulation (Figure 7E).

## DISCUSSION

The conditional *FIR*^+/−^ mouse exhibited prominent *c-myc* upregulation, particularly in the PB among other organs but without a significant pathogenic phenotype (Figure 1). The increased mRNA expression of *FIR*/*FIRΔexon2* was detected in human leukemia cell lines and clinical samples (Figure 1E–G). *FIR*^−/−^ was embryonic lethal in mice before E9.5 (Table 1). *FIR*^+/−^*TP53*^−/−^ mice developed T-ALL (Figure 2) and exhibited an increased incidence of organ or bone marrow invasion (Figure 3). The bodyweight of *FIR*^+/−^*TP53*^−/−^ mice was less than that of *FIR*^+/+^*TP53*^−/−^ mice, and overall survival was reduced in *FIR*^+/−^*TP53*^−/−^ compared with that in *FIR*^+/+^*TP53*^−/−^ mice, presumably due to the rapid progression of T-ALL (Figure 4). The Notch signaling pathway was more activated in sorted CD4^+^CD8^+^ *thymic lymphoma cells isolated from FIR*^+/−^*TP53*^−/−^ (H635) *compared with those from FIR*^+/+^*TP53*^−/−^ (N166) *mice,* as revealed by RNA-sequencing analysis (Figure 5). Quantitative RT-PCR confirmed that c-myc mRNA expression was negatively correlated with *FIR* mRNA expression but positively correlated with *Notch1* mRNA in sorted T-ALL/thymic lymphoma cells (Figure 6). Furthermore, knocking down *TP53* or *c-myc* using siRNA suppressed Notch1 expression; however, TP53 expression was not affected significantly by the enforced expression of FIR/FIRΔexon2 using an adenovirus vector (Figures 7A–D). Thus, alternative splicing of FIR expression increased Notch1 through c-Myc upregulation independent on TP53-pathway (Figure 7E).

DNA damage affects alternative splicing by modulating the elongation activity and/or the phosphorylation status of RNA polymerase II [15,31]. In addition, BLM-induced DNA damage alters FIR splicing, which contributes to the transcriptional upregulation of *c-myc* via dominant negative effect on endogenous FIR [12, 13]. c-Myc accelerates the cell cycle by suppressing p27Kip1 expression, eventually leading to the accumulation of DNA damage. Therefore, FIR splicing is a novel mechanism that connects DNA damage to the regulation of *c-myc*. The sustained c-Myc and Notch1 protein expression level also needs to be regulated by their stability and degradation process. So, how does *FIR* splicing contribute to these processes that are pivotal for T-ALL pathogenesis? One possibility is that *FIR*/*FIRΔexon2* interferes with FBW7 (F-box WD repeat-containing protein 7). FBW7 is a polyubiquitin ligase that acts on Cdc4 phospho-degron (CPD) consensus sequence (−-TP--S/E--)-containing proteins, including c-Myc and Notch1 [32-36]. Therefore, leukemic cells from FBW7-deficient mice exhibited the marked accumulation of Notch-1 and c-Myc proteins, which led to the development of T-ALL [25]. The WD-like motifs (W425 and D399) in the CPD-binding propeller pocket of FBW7 are positioned close to each other in the 3D structure after protein folding, and they potentially interact with FIR-UHM (LNGRWFAGRKVVA) (unpublished data). Purified FIRΔexon2 exhibited a higher binding affinity for Skp1-FWB7 than did FIR in a HiTrap Ni affinity column [37], suggesting that FIRΔexon2 may interact with FBW7 (data not shown). Therefore, FIRΔexon2 may sustain c-Myc protein levels post-transcriptionally by inhibiting the FBW7-mediated pathway. This situation is further complicated because the dimerization site of FIR (PUF60), the RRM2 domain, is not affected by the splicing of exon 2 or 5 in terms of FBP/FIR/FIRΔexon2/PUF60/SAP155 interaction [38, 39]. The difference of the FBP/FIR/FIRΔexon2/PUF60/SAP155 interaction or regulation among organs also needs to be revealed in carcinogenesis in further study.

The KRAS G12D and Notch1 mutations contribute cooperatively to the leukemogenic transformation of normal T-cells in mouse models [40]. Notch1 mutations, which activate *c-myc* transcription, were identified in > 50% of T-ALL cases [41-43], suggesting that upregulation of Notch1 or c-Myc phosphorylation occurred via the EGFR/KRAS/MEK/ERK pathway [37]. How does the splicing of FIR affect Notch1 or c-Myc phosphorylation? Phosphorylated-ERK (p-ERK) is a substrate of protein phosphatase 1 (PP1). Both Ad-FIR and Ad-FIRΔexon2 activated p-ERK but did not affect PP1 in Jurkat and HeLa cells (data not shown). Ad-FIRΔexon2, but not Ad-FIR, elevated pSer62-c-Myc expression much more than expected by *c-myc* mRNA level in HeLa cells [16]. Therefore, FIRΔexon2 sustains c-Myc activation via both transcriptional and post-transcriptional mechanisms. Notably, FLAG-tagged FIR or FIRΔexon2 were co-immunoprecipitated with TOPOII-alpha, SAP155, TRRAP, and filamine-A in pull-down assays [14, 18, 19]; these proteins contain the GKKRVRWADLE sequence, which is specific for interactions with PP1 [44]. Because PP1 inhibits the RAS/BRAF/MEK/ERK pathway [45] and also regulates c-Myc phosphorylation at Ser-62 (pSer62-c-Myc) [46], FIRΔexon2 may interfere with the function of PP1. However, further studies are required to reveal the detailed mechanism behind this potential effect.

Finally, five individuals had *de novo* interstitial 8q24.3 (chr8: 144,868,670–144,933,911; USCS Genome Browser hg19, http://genome.ucsc.edu.) deletions ranging from 65 kb to 1 Mb on the chromosome that encodes *Scrib* [scribbled homologue (MIM607733)], *FIR* (PUF60), and *NRBP2* (nuclear receptor binding protein 2) [29]. Patients with 8q24.3 deletions showed clinical phenotypes associated with coloboma, congenital heart defects, limb abnormalities, psychomotor retardation, and convulsions; however, no hematologic malignancies or lymphoma have been described [30]. Detecting FIR/FIRΔexon2 for diagnosis, or the use of specific antibodies against FIRΔexon2 or chemicals that inhibit the FIR/FIRΔexon2/SAP155 interaction may have clinical applications in T-ALL.

Together, these data suggest that the alternative splicing of FIR may link DNA damage to *c-myc* regulation. Haploinsufficiency of the *c-myc* transcriptional repressor *FIR* and the *FIR*^+/−^*TP53*^−/−^ genotype in mice potently promoted the progression of T-ALL/lymphoma, at least in part by activating the Notch signaling pathway with *c-myc*/c-Myc upergulation. The alternative splicing of *FIR* contributes to not only colorectal carcinogenesis but also leukemogenesis.

## MATERIALS AND METHODS

### Human leukemia samples

Human leukemia, control or adult healthy volunteer samples were obtained from Chiba University Hospital (adult patients) with written informed consent (Table S1).

### Generation of animals and ethical approval

C57BL/6NCrSlc mice were obtained from Japan SLC. Mice were bred and maintained in the animal research facility of the Graduate School of Medicine, Chiba University (Chiba, Japan) in accordance with institutional guidelines. This study was approved by the institutional review committees of Chiba University. All experiments using mice received approval from the Chiba University Administrative Panel for Animal Care. Littermates were used as controls in all experiments.

### Constructing the *FIR*-targeting vector

Construct of FIR targeting vector was indicated (Figures 1 and S1).

### Homologous recombination of the *FIR*-targeting vector in ES cells

First, FIR targeting vector was injected by electroporation for homologous recombination ES cells into (C57BL/6) to prepare genetically modified ES cells (C57BL/6). 23 (clone nos. 26, 29, 31, 45, 84, 105, 112, 114, 117, 125, 145, 146, 172, 176, 178, 179, 185, 188, 191, 238, 244, 245, and 265) of 279 clones were identified in which the FIR-targeting vector had integrated in the chromosomes of ES cells using PCR (Figure S5A). The LoxP site of the integrated FIR-targeting vector was confirmed using PCR with suitable primers (Figure S5B). The integration of the FIR-targeted allele into the genome was confirmed using Southern blotting with 5′ and 3′ probes (Figure S5C). The FIR genomic sequence located between the loxP sites was excised using Cre-recombinase (Figure S5D). After verifying that the ES cells had integrated the FIR-targeting allele successfully using PCR and Southern blotting, the ES cells were microinjected into blastocysts from BALB/c mice. The resulting blastocysts were inoculated into the uterus of ICR mice (Table S2). Male FIR^fl/+^ chimeric mice (chimera mouse) were cross-fertilized with C57BL/6 female mice to obtain F1 FIR heterozygous mice.

### Generating inducible FIR heterozygous knockout mice, *FIR*^+/−^

ES cells were purchased from DS Pharma Biomedical Co., Ltd. (Osaka, Japan). The FIR-targeting vector was prepared from Bac clone using PCR, and the FIR heterozygous knockout mice were prepared using the Cre-loxP system in C57BL6 mice [47] (Unitech Co., Ltd, Chiba, Japan) (Figure S1B). The primers and probes used to prepare FIR heterozygous knockout mice are shown in Figure 1A and Table S2. To assess the function of FIR in hematopoiesis, *FIR* was deleted conditionally by crossing *FIR*^fl/+^ mice with *CAG-Cre* transgenic mice, which express *Cre* ubiquitously (*FIR*^+/−^) (Figure S3). The efficient deletion of *FIR* in fetal cells from *FIR*^+/−^ mice was confirmed using genomic PCR (Figure 1, primers loxP_F1, forward; and loxP_R2; reverse; Figure S6). The number of chimeric mice, born from clone number 26, 105 and 145 were indicated (Table S2). Genetically modified ES cells (C57BL/6) were microinjected into the blastocysts of BALB/c mice. Among the 23 positive clones that contained the FIR-targeting vector, clones 26, 105, and 145 (Figure S6C) were microinjected into the blastocyst cavity of BALB/c mice and were then transplanted into the uterus of pseudo-pregnant ICR mice. Seven chimeric mice were obtained successfully (Figure S6A and Table S2). Cross-fertilization was performed between male FIR^fl/+^ (C57BL/6) and female C57BL/6 mouse to obtain the sperm carrying the FIR^fl/+^ genome (Figure S6B). Finally, FIR^fl/+^ mice were cross-fertilized with CAG-Cre transgenic mice to obtain FIR^fl/+^/Cre(+), which were conditionally inducible FIR heterozygous knockout (*FIR*^+/−^) mice (Figure S6C). FIR heterozygous knockout mice were confirmed using the Cre-LoxP system (Figure S6D).

### Southern blotting of genomic DNA

Three probes were prepared and used for Southern blotting. The 5′-probe (new probe) was located upstream of the short arm in the neomycin gene, and the 3′-probe was located downstream of the long arm (Figure S1). The primers and qRT-PCR conditions used for Southern blotting are shown in Table S5A.

### Registration of *FIR* heterozygous knockout mice [FIR^fl/+^/Cre(+)]

FIR heterozygous knockout mice were established, registered, and made available at the National Institute of Biomedical Innovation (http://animal.nibio.go.jp/j_FIR.html) and the experimental animal division of the RIKEN Bioresource Center, Japan (RBRC No. RBRC05542; http://www2.brc.riken.jp/lab/animal/search.php). Briefly, two loxP sites were inserted upstream of *FIR* exon 3 and downstream of exon 5, respectively, and a PGK-neo cassette and a loxP site was inserted downstream of exon 5. *FIR*-deficient mice could be generated by crossing with tissue-specific Cre mice to give *Mus musculus* C57BL/6-FIR<tm1>/CU.

### TP53-null mice; *TP53*^−/−^

The p53-null mice (*TP53*^−/−^) were purchased from RIKEN BRC (Bio-Resource Center, Tsukuba, Japan; BRC_No 01361, strain name C57BL-p53+/−).

### CAG-Cre-transgenic mice

These mice were a gift from Dr T. Miki [47].

### Measuring the bodyweight and survival curves of mice

The bodyweight of all mice was measured twice a week after the age of 7 weeks. The nose-to-anus length was also measured (Figure S2).

### Immunocytochemistry

Cancer cells were prepared for immunocytochemistry as described previously [8].

### Flow cytometry and cell sorting

The antibodies used for immunostaining and flow cytometry are listed in Table S6. Flow cytometry and cell sorting were performed as described previously [48, 49]. Briefly, linage surface marker antibodies for thymocytes, splenocytes, peripheral blood cells, and bone marrow were Gr-1 (Ly-G6, bone-marrow derived cells), Mac-1 (CD11b, granulocyte, macrophage, etc), B220, CD4 (helper/induced T cell marker), CD8α (cytotoxic T cell marker), CD45.2 (Leukocyte common antigen). Dead cells were eliminated by staining with propidium iodide (1 μg/mL; Sigma-Aldrich). After cell surface staining, intracellular staining was performed using a FITC-conjugated anti-c-Myc antibody. Intraprep^TM^ (Beckman Coulter) was used for fixation and permeabilization. All flow cytometric analyses and cell sorting were performed on FACSAria II or FACSCanto II (BD Biosciences) and analyzed using FACSDiva software (BD Biosciences) and Flowjo (Tree Star, Ashland, OR, USA).

### siRNA

*c-myc* and *TP53* siRNA duplexes were purchased from Sigma-Aldrich (Tokyo, Japan). The transient transfection of siRNAs was performed using Lipofectamine 2000 (Invitrogen) according to the manufacturer's instructions. The transfected cells were cultured for 48–72h at 37°C in a CO_2_ incubator. The target sequences for the siRNAs are listed in Supplementary Table 4.

### Bleomycin treatment

The DNA-damaging agent bleomycin was purchased from Sigma-Aldrich (sulfate powder from *Streptomyces verticillus*; Tokyo, Japan; Lot no. BCBG6499V; PCode, 101203713), dissolved in distilled H_2_O at a concentration of 5 mg/mL, and stored at −20°C. HLF cells were seeded in 6-well plates and incubated at 37°C/5% CO_2_ until confluent (approximately 24 h). Immediately before drug treatment, the media were removed and replaced with fresh culture media. Cells were treated with 30 μg/mL bleomycin alone or co-treated with adenovirus vectors.

### Quantitative real-time PCR (qRT-PCR)

Total RNA was isolated using TRIzol LS solution (Invitrogen) and reverse-transcribed using the ThermoScript RT-PCR system (Invitrogen) with oligo-dT primers. qRT-PCR was performed using an ABI Prism 7300 Thermal Cycler (Applied Biosystems) with FastStart Universal Probe Master (Roche) and Universal Probe Library (Roche). Primers and probes for mouse were listed (Table S5B).

### FIR and FIRΔexon2 adenovirus vectors

FIR and FIRΔexon2 adenovirus vectors were prepared as described previously [18].

### Protein extraction and western blotting

Culture media were removed and the cells were washed twice with cold (4°C) PBS, lysed with 1:20 β-mercaptoethanol and 2x sample buffer, and incubated at 100°C for 5 min. Whole cell lysates were assayed for protein content (Bio-Rad, Hercules, CA, USA), and 10 μg protein samples were separated using SDS-PAGE on 7.5% and 10%–20% XV PANTERA gels. They were then transferred to polyvinylidene fluoride membranes using a tank transfer apparatus, and the membranes were blocked with 0.5% skimmed milk in PBS overnight at 4°C. Membranes were incubated with primary antibodies for 1 h at room temperature, followed by three 10-min washes with PBS/0.01% Tween 20. Membranes were then incubated with secondary antibodies, followed by three 15-min washes with PBS/0.01% Tween 20. Details of the antibodies used in this study are listed in Table S6. Antigens on the membranes were detected using enhanced chemiluminescence detection reagents (GE Healthcare UK Ltd., Buckinghamshire, UK).

### RNA-sequencing

Total RNA was extracted form sorted CD4^+^CD8^+^ and CD4^low+^CD8^+^ thymic lymphoma cells from *FIR*^+/−^*TP53*^−/−^ and *FIR*^+/+^*TP53*^−/−^ mice using TRIzol. The RNA quality was analyzed using a 2100 Bioanalyzer system (Agilent, Santa Clara, CA) to confirm that their RINs (RNA integrity numbers) were > 7.0. RNA-seq was performed to analyze genome-wide gene expression levels. Specifically, RNA-seq libraries were prepared using a TruSeqStranded mRNA LT Sample Prep Kit (Illumina, San Diego, CA) followed by sequencing using a HiSeq1500 genome sequencer (Illumina), according to the manufacturer's protocol. The gene expression levels in *FIR*^+/−^*TP53*^−/−^ mice were compared with those in *FIR*^+/+^*TP53*^−/−^ mice, and the top 100 upregulated genes (Table S2C) were analyzed using KEGG (Kyoto Encyclopedia of Genes and Genomes) software (http://www.genome.jp/kegg/). Signaling pathways with a FDR (false discovery rate) < 1.0 were selected as significantly activated pathways.

### Statistical analysis

The expression of SAP155 and FIR was compared in the lungs, intestine, heart, kidney, and liver of FIR heterozygous knockout adult mice and 14-day-old fetal mice (E14) using Student's *t*-tests and the Wilcoxon test. The WBC, RBC, and platelet counts, organs' weight curve, and the ratio of *FIRΔexon2*/*FIR* mRNA were analyzed statistically using Student's *t*-tests. Overall survival curves were generated using the Kaplan–Meier method and analyzed statistically using log-rank tests. Statistical analyses were performed using **GraphPad Prism version 6.0** for Windows (GraphPad Software, San Diego, CA, USA).

### Accession number and genetic information of FIR genome

Ensemble NM_014281.

## SUPPLEMENTARY MATERIAL, FIGURES AND TABLES


















