# Supplementary material for: Haploinsufficiency of the c-myc transcriptional repressor FIR, as a dominant negative-alternative splicing model, promoted p53-dependent T-cell acute lymphoblastic leukemia progression by activating Notch1
Source: Oncotarget. 2014 Dec 31;6(7):5102–17. doi: 10.18632/oncotarget.3244 (PMC4467136; doi:10.18632/oncotarget.3244)
Supplement: Supplementary file 1 [file oncotarget-06-5102-s001.pdf]

Haploinsufficiency of the *c-myc* transcriptional repressor *FIR*, as a dominant negative-alternative splicing model, promoted p53-dependent T-cell acute lymphoblastic leukemia progression by activating Notch1

Supplementary Material

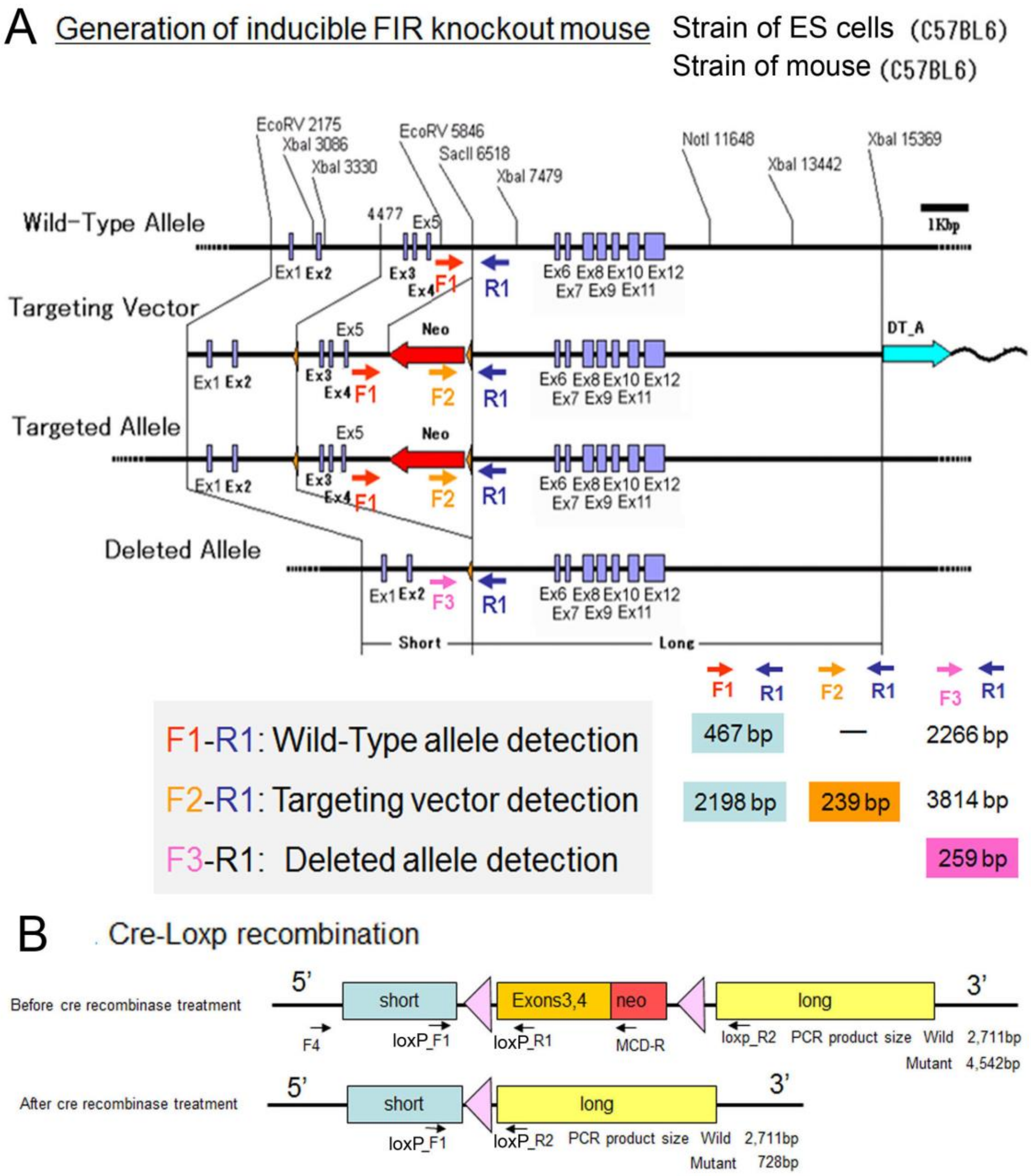

Figure S1: Construction of FIR targeting vector.

(**A**) Conditionally inducible FIR hetero knockout mice were established by Cre-LoxP system. The

design of wild-type allele, targeting vector, targeted allele and deleted allele of FIR was shown. FIR targeting vector was prepared by PCR from Bac clone and FIR hetero knockout mouse was prepared by Cre-loxP system in C57BL6 strain (UNITECH Co., Ltd, Chiba, Japan). Briefly, for the preparation of genetically modified ES cells, FIR targeting vector was electroporated into ES cells of C57BL/6 mouse. (**B**) After checking ES cells by PCR and southern blot analysis that were integrated FIR targeting allele successfully by homologous recombination, the ES cells were injected into blastocysts from BALB/c mouse, and then those blastocysts were inoculated into the uterus of ICR mouse. FIR (flox/+) chimera mouse (chimera mouse) were cross fertilized with C57BL/6 mouse to obtain F1 FIR hetero mouse. FIR targeted allele was confirmed to be integrated in the genome by PCR with suitable primers and southern blot analysis with 5' and 3' probes. FIR genome sequence between the LoxP sites were excised by Cre-recombinase treatment. Primer sequences are indicated in table S1.

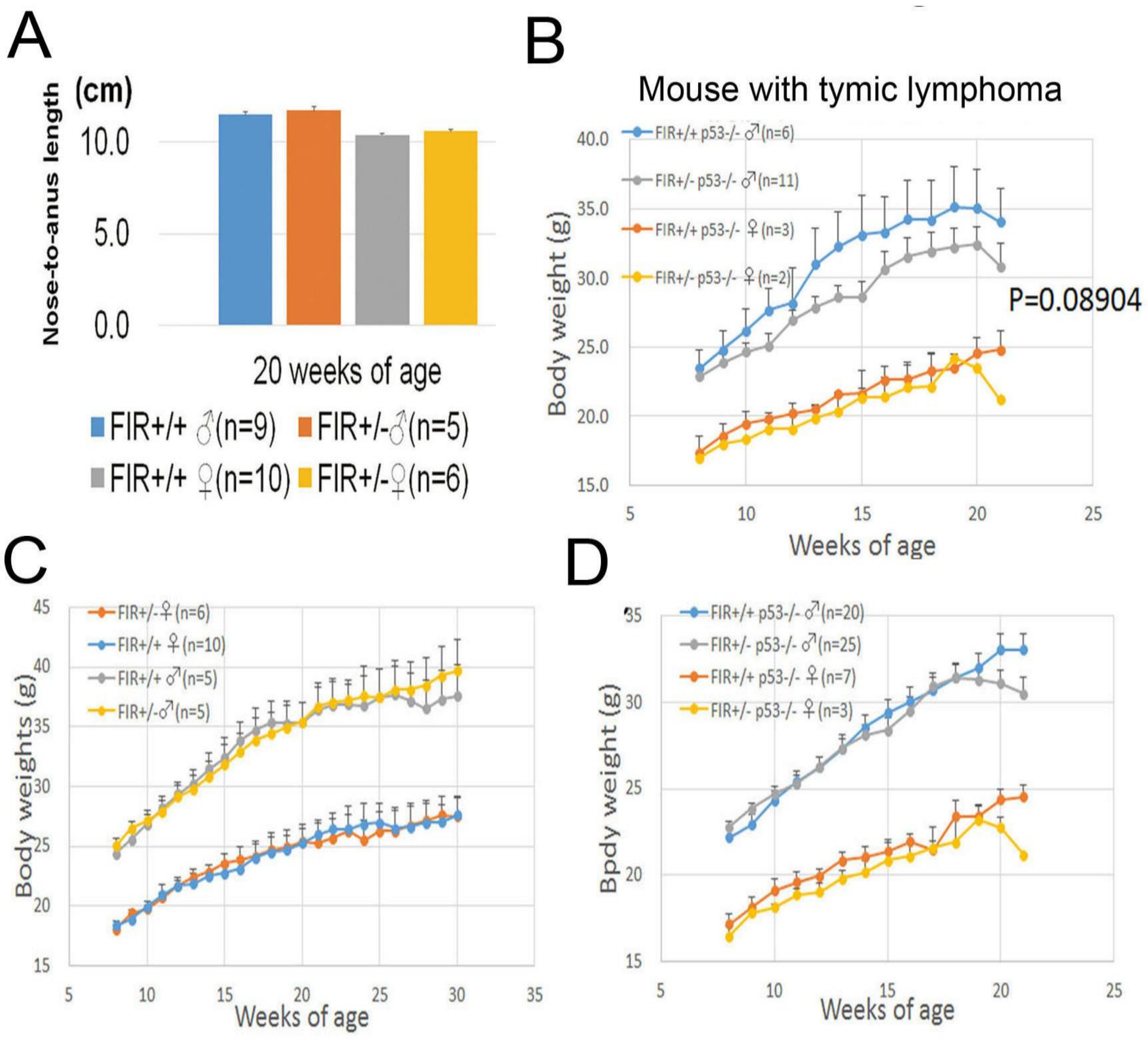

Figure S2: Nose-to-anus length and body weight of mice examined in this study.

(A) Nose-to-anus length and body weight showed no significant difference between *FIR*$^{+/+}$ and *FIR*$^{+/-}$ mouse. Body weight curves of moue, male or female, with **(B)** thymic lymphoma**, (C)** *FIR*$^{+/+}$ or *FIR*$^{+/-}$**, (D)** *FIR*$^{+/-}$*p53*$^{+/+}$, *FIR*$^{+/+}$*p53*$^{+/+}$, *FIR*$^{+/-}$*p53*$^{-/-}$, or *FIR*$^{+/+}$*p53*$^{-/-}$ mice**.**

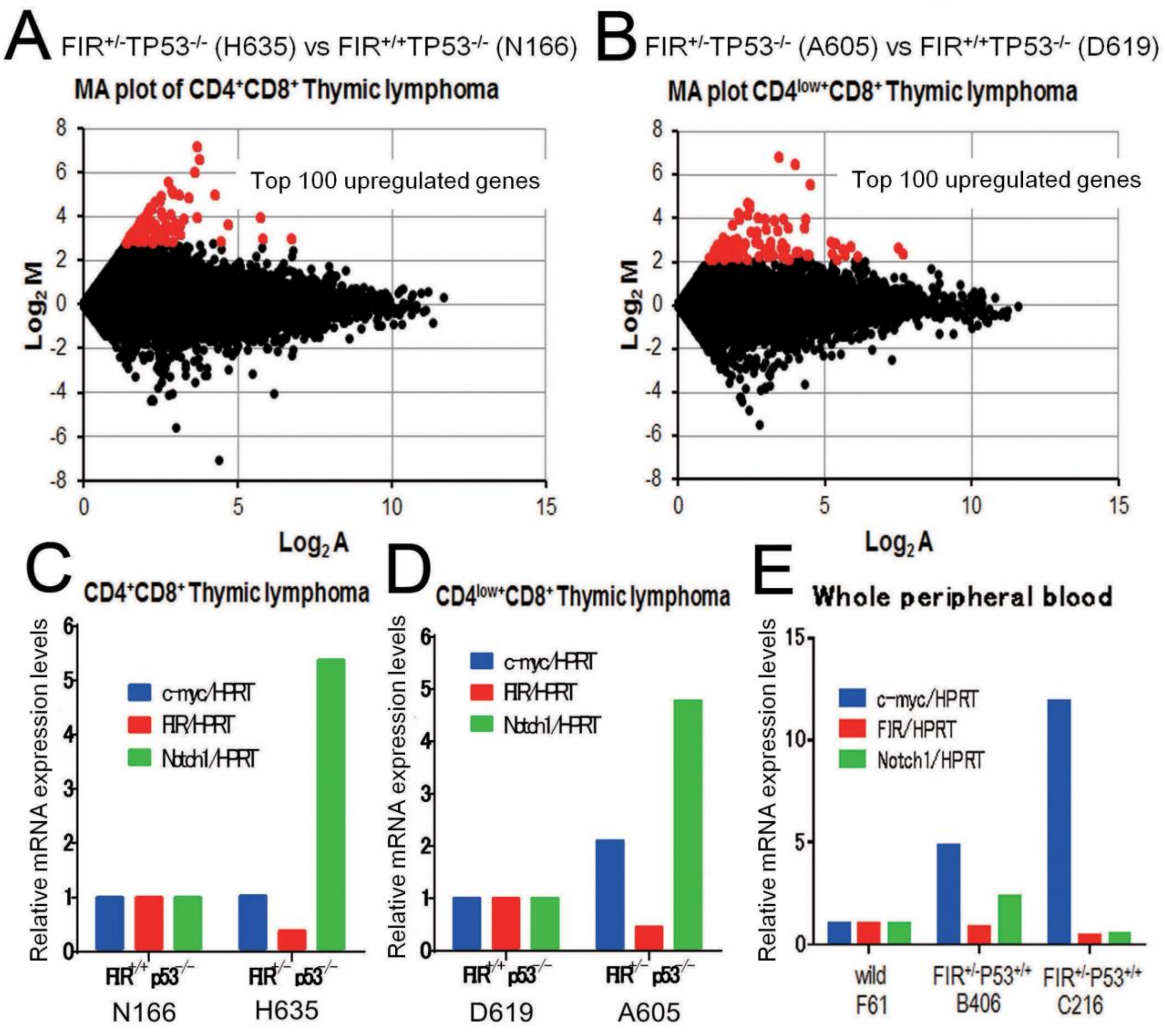

Figure S3: Gene expression analyses. (A) Gene expression levels of *c-myc*, *FIR*, *Notch1* and *Fbw7* were analyzed by real-time RT-PCR and normalized to that of *HPRT*. In $CD4^{low+}CD8^{+}$ thymic lymphoma cells, *c-myc* and *Notch1* were upregulated in $FIR^{+/-}TP53^{-/-}$ mice compared with $FIR^{+/+}TP53^{-/-}$ mice. **(B)** Using RNA-seq data, gene expression levels of $CD4^{+}CD8^{+}$ and $CD4^{low+}CD8^{+}$ thymic lymphoma cells in $FIR^{+/-}TP53^{-/-}$ mice were compared with those in $FIR^{+/+}TP53^{-/-}$ mice. Upregulated (*red dots*) and downregulated (*blue dots*) genes were identified, and top 100 upregulated genes were analyzed by KEGG pathway analysis (Fig 5, Fig S5, table S2). **(C)** qRT-PCR analysis of relative mRNA expression of *c-myc* (blue), FIR (red) and Notch1

(green) in sorted $CD4^{+}CD8^{+}$ thymic lymphoma cells of $FIR^{+/-}TP53^{-/-}$ (A605) and $FIR^{+/+}TP53^{-/-}$ (D619) mouse, and in sorted $CD4^{low+}CD8^{+}$ thymic lymphoma cells of $FIR^{+/-}TP53^{-/-}$ (A605) and $FIR^{+/+}TP53^{-/-}$ (D619) mouse **(D).** **(E)** qRT-PCR analysis of relative mRNA expression of *c-myc* (blue), FIR (red) and Notch1 (green) in the whole peripheral blood of wild type (F61) and $FIR^{+/-}p53^{+/+}$ mice (B406 and C216).

A Tight junction-related genes (*Pard3, Prkch, Amtl1, Ctnna1, Csda, Mllt4*: p=6.95 x 10^{-4}) were also more activated *in FIR$^{+/-}$TP53$^{-/-}$ (H635) than in FIR$^{+/+}$TP53$^{-/-}$ (N166) mouse.*

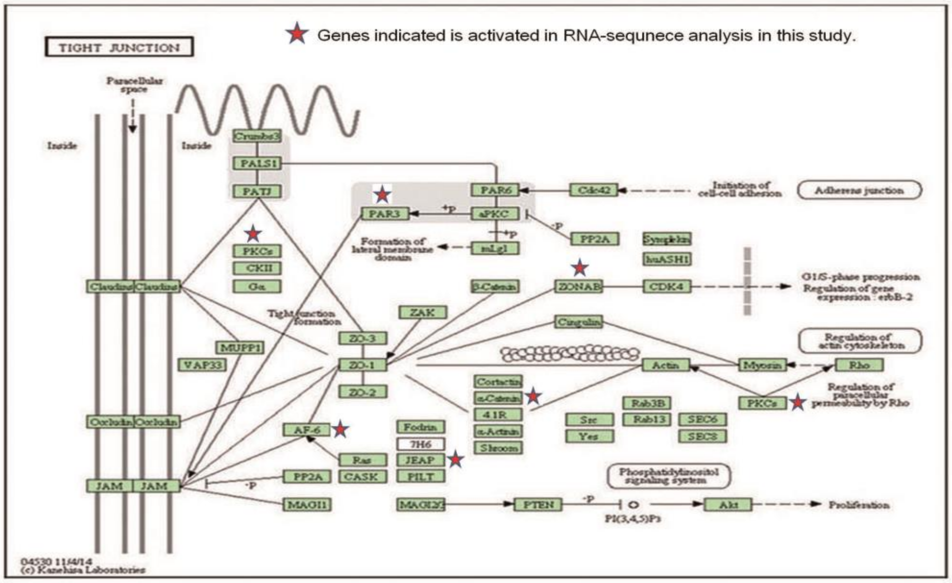

B Focal adhesion pathway (*Igf1r, Lamb2, Jun, Pdgfrb, Pik3r5, Lamc1, Vcl*: p=6.69 x 10^{-4}) was activated in FIR$^{+/-}$TP53$^{-/-}$ (A605) than in FIR$^{+/+}$TP53$^{-/-}$ (D619) mouse.

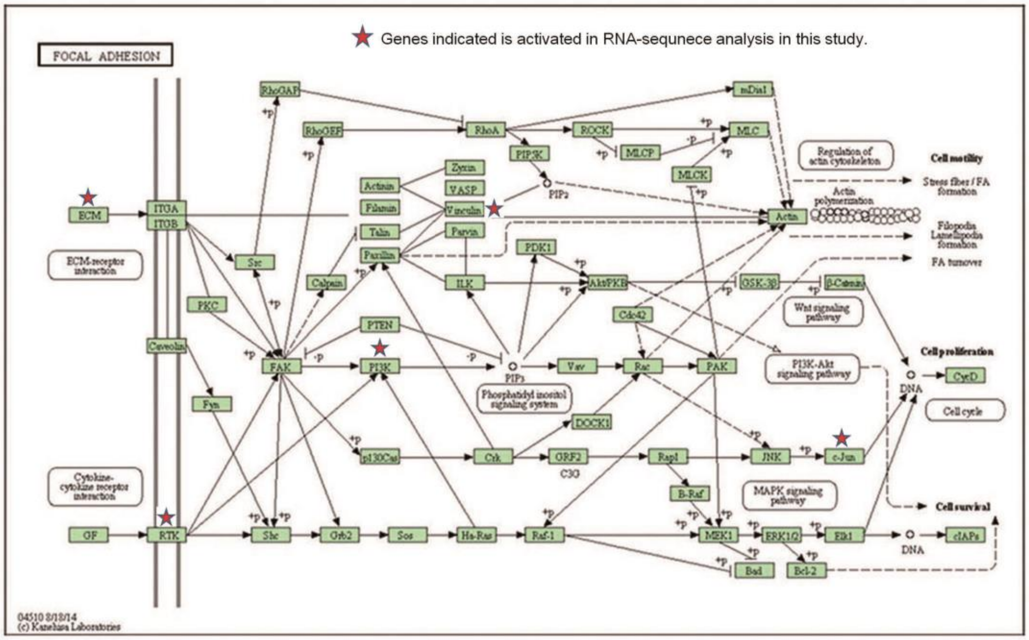

Figure S4: Pathway KEGG pathway analysis activated in RNA-sequence analysis.

(A) Tight junction-related genes (*Pard3*, *Prkch*, *Amtl1*, *Ctnna1*, *Csda*, *Mllt4*: p=6.95 x 10^{-4}) were

also *more* activated in $FIR^{+/-}TP53^{-/-}$ *(H635)* than in $FIR^{+/+}TP53^{-/-}$ *(N166)* mouse. **(B)** In sorted $CD4^{low+}CD8^{+}$ thymic lymphoma cells, focal adhesion pathway (*Igf1r, Lamb2, Jun, Pdgfrb, Pik3r5, Lamc1, Vcl*: p=6.69 x 10^{-4}) was activated *in* $FIR^{+/-}TP53^{-/-}$ (A605) than in $FIR^{+/+}TP53^{-/-}$ (D619) mouse (cf. Table S2A). Top 100 upregulated genes in sorted $CD4^{low+}CD8^{+}$ thymic lymphoma cells of $FIR^{+/-}TP53^{-/-}$ (A605) or $FIR^{+/+}TP53^{-/-}$ (D619) (Table S2C) were listed.

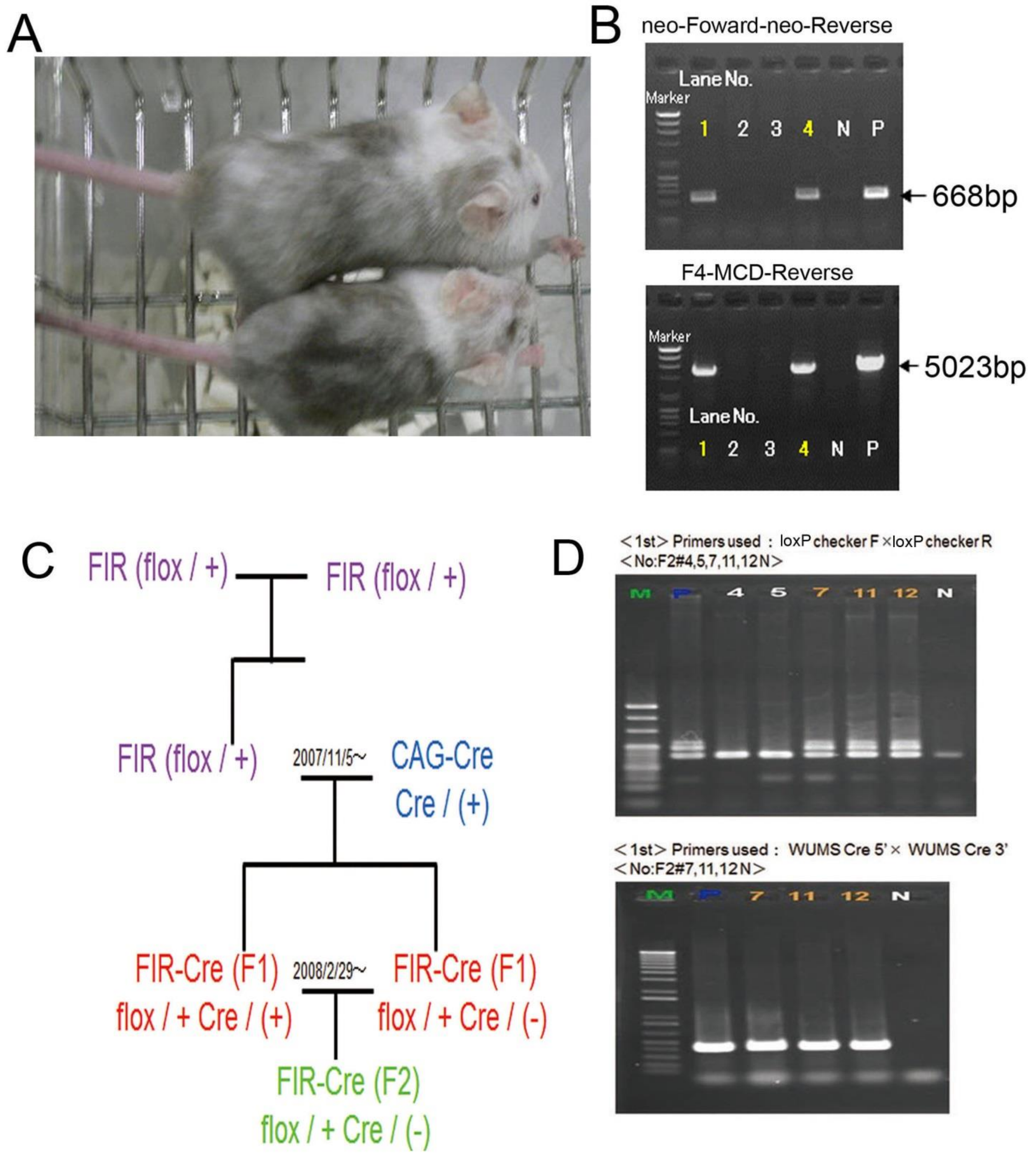

Figure S5: Checking of ES cells whether *FIR* targeting vector is integrated.

(A) FIR targeting vector was injected into the ES cells from C57BL/6 mouse by electroporation.

23 (clone no. 26, 29, 31, 45, 84, 105, 112, 114, 117, 125, 145, 146, 172, 176, 178, 179, 185, 188, 191, 238, 244, 245 and 265) out of 279 clones were identified that FIR targeting vector was integrated in the chromosome of ES cells. Positive clones were identified by confirming loxP site by PCR **(B)**, southern blot analysis **(C)** and cre recombinase treatment **(D)**.

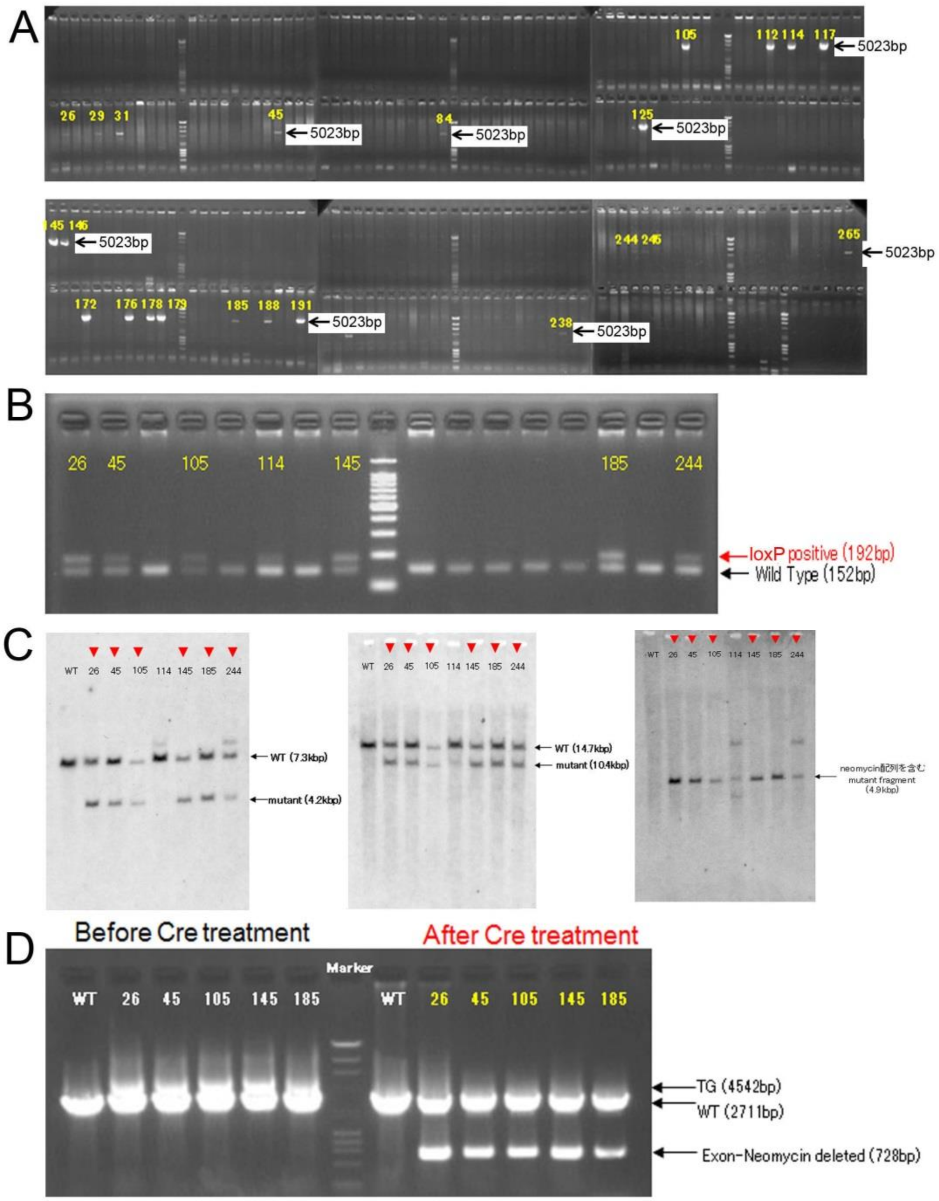

Figure S6: Preparation of *FIR*$^{fl/+}$ chimeric mice

(A) Seven chimera mice were successfully obtained. Male chimeric mice were mated with female wild type C57BL/6 mice. **(C)** Cross-fertilization of $FIR^{fl/+}$ and CAG-Cre mouse to obtain FIR hetero knockout mice: $FIR^{+/-}$. Among 23 positive clones identified having FIR targeting vector, clones 26, 105 and 145 clones were injected into the blastocyst cavity from Balb/c mouse and then inoculated into the uterus of ICR mouse.
